# Supplementary material for: Autologous Micro-Fragmented Adipose Tissue (MFAT) to Treat Symptomatic Knee Osteoarthritis: Early Outcomes of a Consecutive Case Series
Source: J Clin Med. 2021 May 21;10(11):2231. doi: 10.3390/jcm10112231 (PMC8196751; doi:10.3390/jcm10112231)
Supplement: Supplementary file 1 [file jcm-10-02231-s001.zip › jcm-1196508-supplementary.pdf]

Supplementary material

Table S1. Detailed overview of study adverse events (AEs). The parameters 'pain', 'effusion' and 'stiffness' as part of the inflammatory reaction triad were scored as separate adverse events. (MFAT, micro-fragmented adipose tissue; ITB, iliotibial band; OA, osteoarthritis)

Category	AEs	Quantity (%)	Severity	Duration (days)
Liposuction-related	Abdominal pain, swelling or numbness	4 subjects (6.3%)	Moderate: 75%<br>Severe: 25%	33.5 ± 54.6
MFAT-related	Inflammatory triad (at least 1 out of 3)	72 knees (79%)	Mild: 16%<br>Moderate: 52%<br>Severe: 32%	16.6 ± 13.5
	Pain	30 events	Mild: 10%<br>Moderate: 60%<br>Severe: 30%	17.3 ± 12.9
	Swelling	33 events	Mild: 21%<br>Moderate: 49%<br>Severe: 30%	16.8 ± 11.6
	Stiffness	35 events	Mild: 17%<br>Moderate: 46%<br>Severe: 37%	15.7 ± 15.8
Others	Total	7 subjects (10.9%)	Mild: 23%<br>Moderate: 69%<br>Severe: 8%	65.5 ± 103.5
	Subjective Knee instability	3 events	Mild: 67%<br>Moderate: 33%	14.0 ± 0.0
	Tendinopathy (ITB, patella)	3 events	Moderate: 100%	162,7 ± 172.6
	Aching calve muscles	2 events	Moderate: 100%	14.0 ± 0.0
	OA worsening	1 event	Severe: 100%	206.0 ± 0.0
	Stroke	2 events	Moderate: 100%	25.0 ± 0.0
	Gallstones	1 event	Mild: 100%	21.0 ± 0.0
**Total AEs**	/	**119 events in 53 subjects**	**Mild: 16%<br>Moderate: 45%<br>Severe: 39%**	**25.0 ± 42.0**

Table S2. NRS pain, UCLA and EQ-5D index outcomes after MFAT administration. The UCLA and EQ-5D were assessed separately for the unilateral and bilateral injected subjects.

Tx	NRS pain index knee (n=64)	P-value (baseline – Tx)		
Baseline	5.5 ± 2.2	/		
1 month	4.8 ± 2.2	0.0073*		
3 month	3.8 ± 2.4	<0.0001*		
6 month	4.0 ± 2.7	<0.0001*		
12 month	4.2 ± 2.5	0.0002*		
**Tx**	**UCLA Unilateral (n=37)**	P-value (baseline – Tx)	**UCLA Bilateral (n=27)**	P-value (baseline – Tx)
Baseline	6.2 ± 2.2	/	5.8 ± 2.0	/
1 month	/	/	/	/
3 month	6.00 ± 2.1	n.s.	6.1 ± 2.2	n.s.
6 month	6.1 ± 2.1	n.s.	6.4 ± 2.2	n.s.
12 month	6.2 ± 2.1	n.s.	6.5 ± 2.0	n.s.
**Tx**	**EQ-5D Unilateral (n=37)**	P-value (baseline – Tx)	**EQ-5D Bilateral (n=27)**	P-value (baseline – Tx)
Baseline	0.8099 ± 0.0463	/	0.8064 ± 0.0609	/
1 month	/	/	/	/
3 month	0.8429 ± 0.0698	0.0154*	0.848 ± 0.0924	0.0359*
6 month	0.8673 ± 0.0963	0.0007*	0.8509 ± 0.0926	0.0289*
12 month	0.8507 ± 0.084	0.0023*	0.8359 ± 0.082	n.s.
